# Reliability and validity test of VES-13 and analysis of influencing factors for the vulnerable condition of patients with advanced castration-resistant prostate cancer

**DOI:** 10.12669/pjms.37.1.3095

**Published:** 2021

**Authors:** Jia Feng, Qian Sun, Jing Li, Ting-ting Li

**Affiliations:** 1 Jia Feng, Department of Oncology, Affiliated Hospital of Hebei University, Baoding 071000, Hebei, China; 2 Qian Sun, Department of general surgery, Affiliated Hospital of Hebei University, Baoding 071000, Hebei, China; 3 Jing Li, Department of Renal Dialysis, Affiliated Hospital of Hebei University, Baoding 071000, Hebei, China; 4 Ting-ting Li^*^ Department of Cardiothoracic Surgery, Affiliated Hospital of Hebei University, Baoding 071000, Hebei, China

**Keywords:** Advanced castration-resistant prostate cancer, Vulnerability, VES-13 scale, Influencing factors

## Abstract

**Objectives:**

To study the relationship between the reliability and validity of VES-13 and the influencing factors of the vulnerable condition of patients with advanced castration-resistant prostate cancer, so as to provide a reference for the health management and nursing of elderly inpatients.

**Methods:**

By means of convenience sampling, 150 vulnerable patients with advanced castration-resistant prostate cancer who were admitted to the Department of Oncology of the Affiliated Hospital of Hebei University from April 2019 to March 2020 were selected. General data questionnaire, simple mental state checklist, anxiety self-assessment scale, and the vulnerable elders 13 survey (VES-13) were used in this study. The reliability and validity were tested by SPSS 20.0.

**Results:**

For this group of patients, VES-13 Cronbach’s α was 0.832, living function was 0.778, body function was 0.846, and the correlation coefficient between items was between 0.401 and 0.823 (P<0.01); the retest reliability was 0.831 (P<0.05), and the correlation coefficient was0.504 (P<0.05). The average time to fill in each scale was 4.53±1.32 Minutes, and general indicators such as economic status and education level were significantly correlated with vulnerability. The clinical indicators of prostate-specific antigen levels, multiple bone metastases, and bone pain were significantly correlated with vulnerability.

**Conclusion:**

VES-13 scale had high level of reliability and operability, and was straight forward to understand and use; it can be used as an effective tool for the assessment of vulnerability in patients with advanced prostate cancer. The vulnerability of patients with castration-resistant prostate cancer showed significant correlation with economic status, education level, prostate-specific antigen level, multiple bone metastases, bone pain and other indicators. Nursing staff should pay close attention to the relevant factors of inpatients and give targeted advance intervention measures to facilitate rapid recovery of elderly inpatients.

## INTRODUCTION

Vulnerability refers to the decrease in the physiological reserve capacity of multiple systems in the human body, which increases the vulnerability of the human body and reduces their ability to resist stress.^1^ A slight stimulation can reduce the gravitational function and increase the risk of falls and death. 2 With the intensification of population aging in China, vulnerability has gradually become an important issue that threatens the health of the elderly. Coexistence of multiple diseases and mental psychology can affect the occurrence and development of vulnerability.^3^ Prostate cancer is a common urogenital tumor in elderly men, especially for castration-resistant prostate cancer, which has long treatment period, poor treatment effect, high cost, and is often accompanied by bone metastasis and bone pain.^4^ However, this group of people has not received attention in the daily care in hospital. The purpose of this study was to investigate the current status of vulnerable patients with castration-resistant prostate cancer, analyze related factors, and to test the reliability and validity of VES-13, with a view to providing a reference for the screening of such vulnerable patients.

## METHODS

Patients with advanced castration-resistant prostate cancer who were consecutively admitted to the Affiliated Hospital of Hebei University from April 2019 to March 2020 were included in this study. *Inclusion criteria*: (1) age ≥60 years; (2) clear tumor diagnosis (including clinical and pathological diagnosis of prostate biopsy); (3) no mental and cognitive disorder, normal communication ability; (4) informed consent and voluntary participation. *Exclusion criteria:* (1) patients with unclear expressions and unconsciousness; (2) patients withdrew midway; (3) patients with dementia; (4) patients could not complete the assessment for other reasons. Privacy of patients was protected by using anonymous questionnaire which were collected or mailed on the spot; coded questionnaire were reviewed and data were inputted by two staffs.

### Ethical Approval

The study was approved by the Institutional Ethics Committee of Affiliated Hospital of Hebei University, and written informed consent was obtained from all participants.

General questionnaire included age, education level, marital status, monthly income, multiple medications (taking ≥ 5 oral medications at the same time). At the same time, patients’ clinical data such as prostate specific antigen level, bone metastasis, bone pain, kidney function, liver function, volume of prostate, and residual urine, etc. were collected.

### The Vulnerable Elders Survey (VES-13)

The Vulnerable Elders Survey (VES-13) was prepared by American scholars in 2001 and is an effective tool for screening the degree of vulnerability (risk of injury and physical function declining) of the vulnerable elderly. VES-13 can be used in prediction of cancer, heart failure, etc., as well as the vulnerability, negative events, self-care ability, physical and mental function declining, mortality of elderly people with chronic disease.^5^-^6^ The accuracy rate of mortality risk is 32%, the sensitivity is 72.7%, the specificity is 85.9%, and it is accurate rate for those with high functional declining is higher, and Luz LL et al.^7^ has concluded that it also has very good reliability and validity in the general elderly. The overall evaluation value is 88.9%. VES-13 is divided into four dimensions of age, self-assessment health, physical function and living function, a total of 13 items. The higher the score, the more vulnerable the elderly and the greater the risk of injury.

The final scale was equivalent to the source scale as its translation, back translation, language and cultural adjustment, and expert review were completed and revised strictly in accordance with the international standards. The Chinese version of VES-13 included 4 aspects of age, self-assessed health status, activity status, and functional status, with a total of 13 items. Three points or less indicated normal, and ≥3 points indicated vulnerable. According to the clinical characteristics of castration-resistant prostate cancer, it was divided into three levels: <6 was mild vulnerability, six to eight moderate vulnerability, and >8 was severe vulnerability. The scale was straight forward and easy to use.

### Preliminary Experiment

Fifteen Elderly patients were selected for the preliminary experiment. All 15 elderly patients could complete the questionnaire in 5 Minutes (average 4.53±1.32 Minutes). The results showed that Cronbach’s α was 0.847. According to the preliminary experiment, the questionnaire was further improved. The vocabulary in the questionnaire that was difficult for the elderly to understand was revised.

### Statistical Methods

Data was analyzed by SPSS23.0 and AMOS17.0. Reliability was expressed by Cronbach’s α and other coefficients; exploratory factors and confirmatory factors were used to test the validity of VES-13. The total score correlation of items was expressed by Pearson correlation coefficient. The data were compared by t test or analysis of variance. Non-normal distribution was tested by non-parametric test. Logistic regression was used in multivariate analysis. P <0.05 indicated the difference was statistically significant.

## RESULTS

### Validity assessment of VES-13 scale

150 questionnaires were distributed, 147 were recovered, of which 140 were valid. The response rate was 95.2%. 72 people (48%) were 60 to 70 years old, 43 people (29%) were 71 to 80 years old, and 35 people (23%) were 81 years old and above. 68 people (45%) were high school graduated and below, 42 people (28%) were junior college graduated, and 40 people (27%) held a bachelor’s degree or above. 110 people (73%) were married, 7 people (5%) were cohabiting, 11 people (7%) were widowed, and 22 people (15%) were living alone or separated. 93 people’s monthly income <5000, 32 people’s monthly income were 5000~10000, and 25 people’s monthly income >10000. 44 people took multiple drugs, 32 people took 5 drugs orally, and 12 people took more than 5 drugs at the same time. Reliability test and Cronbach’s α coefficient of VES-13: VES-13 was 0.832, living function was 0.778, body function was 0.846, and correlation coefficients between items were between 0.401 and 0.823 (P<0.01); The test-retest reliability was 0.831 (P<0.05), and the correlation coefficient was 0.504 (P<0.05). The results indicated that VES-13 had good reliability and validity, and can be used as an evaluation method for evaluating the vulnerability of patients with castration-resistant prostate cancer.

VES-13 was used to analyze patient’s economic status, education background, marital status, and medications use. The results suggested that age, education background, marital status, and income are significantly correlated with the vulnerability degree of patients with advanced castration-resistant prostate cancer. Old age and high education level indicated severe degree of vulnerability. Individuals living alone had a high proportion of severe vulnerability. In terms of income, low-income people have a high prevalence of vulnerable diseases and a large proportion of severe vulnerability. There was no significant correlation between taking multiple medications and the degree of vulnerability.

Total prostate specific antigen level (TPSA) was positively correlated with the severity of vulnerability; free PSA level was negatively correlated with vulnerability. Bone metastasis was insignificantly correlated with vulnerability, and bone pain was positively correlated with vulnerability. Renal function was not significantly correlated with the degree of vulnerability, and residual urine was positively correlated with the degree of vulnerability (Table-II)

**Table-I:** Evaluation of the relationship between VES-13 phenotype and general information of patients

Items	Mild vulnerability N(%)	Moderate vulnerability N(%)	Severe vulnerability N(%)	Statistical value	p
**Age (yrs)**					
60~70	46(63.8)	17(23.6)	9(12.6)	26.173	0.003
71~80	19(44.2)	17(39.5)	7(16.3)		
≥81	8(22.9)	11(31.4)	16(45.7)		
**Education Level**					
High school and below College	34(50%)	22(32.4)	12(17.6)		
	10(23.8)	14(33.3)	18(42.9)	8.209	0.016
Bachelor’s degree and above	13(32.5)	10(25%)	17(42.5)		
**Marriage**					
Married	80(72.7)	13(11.8)	17(15.5)		
Cohabitation	2(28.6)	0	5(71.4)	11.948	0.017
Widowed	1(9)	5(45.5)	5(45.5)		
Living alone	7(31.8)	3(13.6)	12(54.6)		
**Income (RMB)**					
<5000	26(27.9)	24(25.8)	43(46.3)		
5000~10000	19(59.4)	10(31.3)	3(9.3)	8.89	0.012
<10000	20(80)	3(12)	2(8)		
**Multiple medication**					
Take 5 drugs at the same time More than 5 drugs	15(34.1)	17(38.6)	12(27.3)	1.97	0.181
More than 5 drugs	4(33.3)	5(41.7)	3(25)		

**Table-II:** Analysis of clinical indicators of patients and VES-13 phenotype (r).

VER-13 Degree of vulnerability	TPSA	FPSA	Bone metastasis	Bone pain	Creatinine	Residual urine
Mild	0.131	-0.062	0.130	0.035	0.135	0.976
Moderate	0.252	-0.046	0.129	0.042	0.137	1.373
Severe	0.258	-0.016	0.137	0.065	0.158	1.298

## DISCUSSION

At present, the aging of the population is becoMinutesg increasingly severe.^8^ In particular, there are more and more elderly patients with advanced tumors, resulting a phenomenon of vulnerability that has not received the attention it deserves. The elderly aged above 60 often have cancer accompanied by other diseases such as pain, abnormal movements, etc.^9^ This group of people with reduced physical and mental function and high risk of falling and death are regarded as vulnerable elderly.^10^ Prostate cancer is a common urological tumor in elderly patients, and the incidence increases with age. Most patients eventually converted to castration-resistant prostate cancer after endocrine therapy.^11^ At this point, classic treatments are gradually losing their effects on those patients, and the cancer cases multiple bone metastases and bone pain. In addition, at this stage, patients are switched from endocrine therapy with Minutesimal side effects to high-cost treatment such as chemotherapy and targeted therapy, which requires frequent hospitalization (one to three months).^12^ Therefore, these patients will undergo certain anxiety, personality changes, and vulnerability. One hundred fifty vulnerable patients included in this study who were repeatedly hospitalized accounted for 82.5% (150/183) of patients who were hospitalized with castration-resistant prostate cancer during the same period. This shows that the incidence of these type of patients is very high. Therefore, no matter from the clinical or sociological point of view, this study is of great necessity.

VES-13 is a simple self-assessment screening tool developed in 2001, it has been used to identify the “vulnerable” elderly in the community who are at risk of health deterioration.^13^ It is currently the most widely used assessment tool. Mohile et al.^14^ used VES-13 for the first time in screening of vulnerable elderly tumor patients. The results suggested that it has good predictive validity. Kenig et al.^15^ also suggested that VES-13 has good predictive validity. This study suggested that the reliability test of VES-13 and the Cronbach’s α coefficient of internal consistency within the scale: VES-13 was 0.832, living function was 0.778, body function was 0.846, and the correlation coefficient between items were between 0.401 and 0.823 (P< 0.01); the retest reliability was 0.831 (P<0.05), and the correlation coefficient was 0.504 (P<0.05). The results were similar to those reported before, suggesting that VES-13 was applicable to patients with castration-resistant prostate cancer.^16^-^17^ The time needed to fill out the form was short and the content was easy for patients to understand.

Castration-resistant prostate cancer is the advanced stage of prostate cancer[18]. At this point, patients develop resistance to endocrine therapy (basic treatment for advanced prostate cancer with small side effects and significant efficacy). The treatment plan mostly is expensive, and adopts targeted therapy and chemotherapy.^19^-^20^ Therefore, the incidence of vulnerable patients is extremely high. A total of 150 patients included in this study showed different degrees of vulnerability, accounting for more than 80% of the total number of patients in the same period (82.5%, 150/183). Results indicated that most of these patients were vulnerable. In these patients, as the age increases, the degree of vulnerability increases. Results suggested that 45.7% of patients over 80 years old were severely vulnerable. This study suggested that income was negatively correlated with the degree of vulnerability, suggesting that the cost of treatment has a certain impact on the patient’s mental and cognitive aspects.

Clinical indicators mostly have effect on patients’ PSA level, bone pain, and residual urine volume. The result for this could be as following: 1. PSA directly reflects the quality of tumor control. If PSA level increases, patient’s psychological burden increase accordingly; 2. bone pain and residual urine reflect the patient’s physical comfort. Both bone pain and increased residual urine will aggravate patient’s pain. Therefore, the clinical indicators not only affect the patient’s body, but also bring a certain psychological burden to the patients. To provide patients with clinical indicators of reasonable comfort, while actively reducing the patient’s body pain, to avoid further deterioration of vulnerability. This study has important clinical value for promoting the disease rehabilitation and physical and mental health of patients with castration-resistant prostate cancer.

### Limitations of the Study

Due to the small sample size, it is not possible to deterMinutese whether it is age itself, or whether the length of hospital stay or cost over time was an independent risk factor for vulnerability progress. Further research is needed with large sample size.

## CONCLUSION

1) The VES-13 scale has high reliability and operability, and is straight forward to understand and use; it can be used as an effective tool for the assessment of vulnerability of patients with advanced prostate cancer; 2) The vulnerability of patients with castration-resistant prostate cancer is significantly correlated with age, education background, marital status, and income. Elderly, low-income, high-education, and solitary patients should be regarded as key target for nursing interventions; 3) Prostate-specific antigen levels, bone pain, residual urine and other indicators have a significant correlation with vulnerability. Nursing staff should pay close attention to the clinical related factors of inpatients, and give targeted advance intervention measures to facilitate the rapid recovery of elderly inpatients.

### Authors’ Contributions:

**JF** & **QS:** Designed this study and prepared this manuscript, and are responsible and accountable for the accuracy or integrity of the work. **TTL:** Collected and analyzed clinical data. **JL:** Significantly revised this manuscript.
